# Dog vaccination with EgM proteins against *Echinococcus granulosus*

**DOI:** 10.1186/s40249-018-0425-4

**Published:** 2018-06-13

**Authors:** Zhuang-Zhi Zhang, Gang Guo, Jun Li, Bao-Xin Shi, Li Zhao, Bao-Ping Guo, Xu Zhang, Jun-Wei Wang, Xue-Ting Zheng, Wen-Jing Qi, Li He, Wen-Bao Zhang

**Affiliations:** 1 Xinjiang Veterinary Research Institute, Xinjiang Academy of Animal Sciences, 726 Dongrong Street, North Gaoxin District, Urumqi, 830011 Xinjiang China; 2 State Key Laboratory of Pathogenesis, Prevention and Treatment of High Incidence Diseases in Central Asia, Clinical Medicine Institute, The first Affiliated Hospital of Xinjiang Medical University, Urumqi, 830054 Xinjiang China

**Keywords:** *Echinococcus granulosus*, EgM proteins, Dog vaccine, Adjuvant

## Abstract

**Background:**

Dogs play a pivotal role in the transmission of cystic echinococcosis (CE), a zoonosis caused by the tapeworm *Echinococcus granulosus*. We showed previously that dogs vaccinated with two *E. granulosus* adult-worm specific proteins, EgM9 and EgM123, emulsified with Freund’s adjuvants induced significant protective efficacy in terms of reduction in worm burden and egg production after 45 days post-infection. It was not known whether this protection can be sustained using adjuvants suitable for use in dogs.

**Methods:**

Recombinant EgM9 and EgM123 were mixed with Quil A or ISCOMs for vaccinating dogs. After three vaccine injections, all the dogs were orally challenge-infected with 200 000 protoscoleces of *E. granulosus*. After 45 days of infection, all the dogs were euthanized and necropsied for collecting and counting *E. granulosus* worms. Immunoglobins, including the IgG subclasses IgG1 and IgG2, were detected in the sera of vaccinated dogs by ELISA. To determine whether the protection efficacy could be maintained after 45 days post-infection, we implemented a longevity trial to count eggs in dog faeces for 170 days after infection.

**Results:**

The dogs vaccinated with EgM9 and EgM123 mixed with Quil A and ISCOMs showed similar protective efficacy as the proteins emulsified with Freund’s adjuvants in our previous study in terms of reduction of worms and eggs at 45 days post-infection. The longevity trial showed that EgM9 protein-vaccinated group released lower number of eggs per gram compared with the egg counts in the control dogs during the dog trial study.

**Conclusion:**

EgM9 and EgM123 are thus suitable vaccine candidates against *E. granulosus* infection in dogs.

**Electronic supplementary material:**

The online version of this article (10.1186/s40249-018-0425-4) contains supplementary material, which is available to authorized users.

## Multilingual abstract

Please see Additional file 1 for translations of the abstract into the five official working languages of the United Nations.

## Background

Cystic echinococcosis (CE) is caused by the larval stage of the dog tapeworm *Echinococcus granulosus* and is increasingly recognized as a major public health problem globally [1, 2]. Given the wide endemicity and severity of the disease, the World Health Organisation (WHO) has included CE as one of the 17 neglected diseases in its strategic roadmap for 2020 [3]. However, CE is difficult to control in most continental endemic areas. Dogs are major definitive hosts for *E. granulosus* and play a pivotal role in the transmission of echinococcosis to humans. Vaccination of dogs against *E. granulosus* infection provides an acceptable and cost-effective complementary means to control echinococcosis as there are far fewer dogs than sheep on farms [4, 5]. In our previous studies, we isolated three genes, termed EgM4, EgM9 and EgM123 from mature adult worm of *E. granulosus* [6], and two proteins, EgM9 and EgM123 emulsified with Freund’s adjuvant induced protective efficacy against *E. granulosus* infection in dogs in terms of reduction in worm burden and egg production after 45 days post-infection [7]. However, Freund’s adjuvant is not a suitable adjuvant for vaccination of dogs. In the present study, we used Quil A as adjuvant to confirm the vaccine efficacy of the EgM proteins. We also used commercial ISCOMs as an adjuvant for the EgM protensagainst infection. In addition, we checked *E. granulosus* eggs in the faeces of dogs for 170 days post-infection to test the longevity of protection induced by EgM9 and EgM123.

## Methods

### Dogs for vaccination trials

Dogs were purchased from two resources: 1) Beagles (aged 8–12 months) were purchased from Shanghai and Guangdong Animal Centres; and 2) dogs (local strain, 8-20 months) were from areas in Xinjiang where a hydatid disease control program had been in operation mainly through dosing dogs monthly with praziquantel for a number of years. Dogs without tapeworm infection were selected having been tested used the purging method [8]. In each of the trials, dogs, half male and half female, were randomly divided into each group. The number of dogs in each group are shown in Tables 1, 2 and 3. All the dogs were housed in a quarantine facility at the Veterinary Research Institute, Xinjiang Academy of Animal Sciences, Urumqi, China and were maintained on dog biscuits, and tap water.

**Table 1:** *E. granulosus* worm burdens at day 45 post-infection in Beagles vaccinated with rEgM9/ Quil A adjuvant

Group (protein)	Dog No.	Number of worms						
		Total	≤3 seg	%	≥4 seg	%	Gravid Seg	%
GST controls	71 122	17 154	8025	46.8	7445	43.4	1684	9.8
	71 123	43 725	16 425	37.6	17 833	40.8	9467	21.7
	71 124	14 543	4908	33.7	7452	51.2	2183	15.0
	71 125	34 779	14 792	42.5	15 162	43.6	4825	13.9
	71 126	18 909	9233	48.8	8267	43.7	1409	7.5
	71 127	32 606	16 333	50.1	8867	27.2	7406	22.7
	71 129	19 204	6358	33.1	9912	51.6	2934	15.3
	Average	25 846	10 868	42.0	10 705	41.4	4273	16.5
	*SD*	11 111	4878	–	4120	–	3114	–
	Median	19 204	9233	48.1	8867	46.2	2934	15.3
	IQR	(17 154, 34 779)	(6358, 16 333)	–	(7452, 15 162)	–	(1684,7406)	–
EgM9	71 112	21 008	20 496	97.6	512	2.4	0	0.0
	71 113	9229	8475	91.8	754	8.2	0	0.0
	71 114	9340	4588	49.1	4752	50.9	0	0.0
	71 115	12 824	12 342	96.2	482	3.8	0	0.0
	71 116	1558	1558	100.0	0	0.0	0	0.0
	71 117	28 801	19 651	68.2	8783	30.5	367	1.3
	71 128	30 746	26 083	84.8	4167	13.6	496	1.6
	Average	16 215	13 313	82.1	2779	17.1	123	0.8
	*SD*	10 916	9069	–	3269	–	214	–
	Median	12 824	12 342	96.2	754	5.9	0	0
	IQR	(9229, 28 801)	(4588, 20 496)	–	(482, 4752)	–	(0, 367)	–
	Redu %^a^	33.2	–	–	–	–	100	–
	*P* value^b^	0.128	0.655	–	0.004	–	0.001	–

^a^Compared to those in the GST group. Redu, reduction = (worm burden median in GST-worm burden median in the experiment group)/worm burden median in GST group × 100%. ^b^The Mann-Whitney *U* test was used to compare the worm burden median. *P* value less than 0.05 means that reduction is significant between GST and experimental groups using median analysis. The meaning of ^a, b^is the same in the following tables

**Table 2:** *E. granulosus* worm burdens at day 45 post-infection with in dogs (local Xinjiang breed) vaccinated with rEgM4 and rEgM123 proteins mixed with Quil A

Group (protein)	Dog No.	Number of worms						
		Total	≤3 seg	%	≥4 seg	%	Gravid Seg	%
GST	815	10 228	1063	10.4	9165	89.6	0	0.0
	817	17 897	8103	45.3	7528	42.1	2266	12.7
	821	22 505	677	3.0	21 828	97.0	0	0.0
	822	27 892	11 151	40.0	9529	34.2	7212	25.9
	829	42 724	18 311	42.9	12 532	29.3	11 881	27.8
	831	26 808	18 762	70.0	8046	30.0	0	0.0
	833	9484	839	8.8	8645	91.2	0	0.0
	834	34 147	15 618	45.7	12 085	35.4	6444	18.9
	837	11 053	927	8.4	10 126	91.6	0	0.0
	841	47 079	19 658	41.8	17 424	37.0	9997	21.2
	842	31 342	20 582	65.7	4099	13.1	6661	21.3
	843	13 535	6178	45.6	4237	31.3	3120	23.1
	Average	24 558	10 156	41.4	10 437	42.5	3965	16.1
	*SD*	12 708	8174	–	5083	–	4319	–
	Median	24 657	9627	39.0	9347	37.9	2693	10.9
	IQR	(11 674, 33 446)	(961,18 649)		(7787, 2309)		(0,7074)	–
EgM4	813	34 908	1805	5.2	25 050	71.8	8053	23.1
	816	0	0	0	0	0.0	0	0.0
	818	11 624	3256	28.0	6271	53.9	2097	18.0
	819	0	0	0	0	0.0	0	0.0
	820	14 938	12 469	83.5	1696	11.4	773	5.2
	826	9939	7982	80.3	1103	11.1	854	8.6
	832	0	0	0	0	0.0	0	0.0
	838	1796	1796	100.0	0	0.0	0	0.0
	840	39 773	27 124	68.2	8897	22.4	3752	9.4
	Average	18 830	9072	48.2	7170	38.1	2588	13.7
	*SD*	15 056	9779	–	9399	–	2982	–
	Median	9939	1805	18.2	1103	11.1	773	7.8
	IQR	(0, 24 923)	(0, 10 225)	–	(0, 7584)	–	(0, 2925)	–
	Redu%^a^	59.7	81.3	–	88.2	–	71.3	–
	*P* value^b^	0.069	0.219	–	0.013	–	0.442	–
EgM123	814	128	128	100.0	0	0.0	0	0.0
	823	1075	1075	100.0	0	0.0	0	0.0
	824	0	0	0	0	0.0	0	0.0
	825	0	0	0	0	0.0	0	0.0
	827	3198	3198	100.0	0	0.0	0	0.0
	828	3494	3494	100.0	0	0.0	0	0.0
	835	0	0	0	0	0.0	0	0.0
	836	408	408	100.0	0	0.0	0	0.0
	839	0	0	0	0	0.0	0	0.0
	Average	1661	1661	100.0	0	0.0	0	0.0
	*SD*	1580	1580	–	0	–	0	–
	Median	128	128	–	0	–	0	–
	IQR	(0, 2137)	(0, 2137)	–	(0, 0)	–	(0, 0)	–
	Redu%^a^	99.5	98.7	0	100	–	100	–
	*P* value^b^	0.000	0.002	–	0.000	–	0.023	–

^a^Compared to those in the GST group. Redu, reduction = (worm burden median in GST-worm burden median in the experiment group)/worm burden median in GST group × 100%. ^b^The Mann-Whitney *U* test was used to compare the worm burden median. *P* value less than 0.05 means that reduction is significant between GST and experimental groups using median analysis. The meaning of ^a, b^is the same in the following tables

**Table 3:** *E. granulosus* worm burdens at day 45 post-infection with in dogs (local Xinjiang breed) vaccinated with rEgM9 and rEgM123 proteins coupled with ISCOMs

Group (protein)	Dog No.	Number of worms						
		Total	≤3 seg	%	≥4 seg	%	Gravid Seg	%
GST control	802	24 618	24 618	100.0	0	0.0	0	0.0
	810	15 273	15 273	100.0	0	0.0	0	0.0
	811	2784	2784	100.0	0	0.0	0	0.0
	1313	41 288	22 857	55.4	18 431	44.6	0	0.0
	1314	11 547	7362	63.8	4185	36.2	0	0.0
	Average	19 102	14 579	76.3	11 308	59.2	0	0.0
	*SD*	14 669	9500	–	10 073	–	0	–
	Median	15 273	15 273	–	0	–	0	–
	IQR	(7166, 32 953)	(5073,23 738)	–	(0, 11 308)	–	(0,0)	–
EgM9	804	41 573	6214	14.9	35 359	85.1	0	0.0
	808	59	8	13.6	51	86.4	0	0.0
	848	13 262	13 262	100.0	0	0.0	0	0.0
	849	2933	2933	100.0	0	0.0	0	0.0
	Average	14 457	5604	38.8	8853	61.2	0	0.0
	*SD*	18 946	5700	–	24 967	–	0	–
	Median	8098	4574	–	26	–	0	–
	IQR	(778, 34 495)	(739, 11 500)	–	(0, 26 532)	–	(0,0)	–
	Redu%^a^	47.0	59.3	–	-100	–	0	–
	*P* value^b^	0.624	0.142	–	0.788	–	1.000	–
EgM123	803	5729	5729	100.0	0	0.0	0	0.0
	806	12 058	12 058	100.0	0	0.0	0	0.0
	812	28	28	100.0	0	0.0	0	0.0
	847	1653	301	18.2	1352	81.8	0	0.0
	850	23	23	100.0	0	0.0	0	0.0
	Average	3898	3628	93.1	270	34.7	0	0.0
	*SD*	5123	5303	–	604	–	0	–
	Median IQR	1653 (26, 8894)	301 (26, 8894)	–	0 (0,676)	–	0 (0,0)	–
	Redu%^a^	89.2	98.0	–	0	–	0	–
	IQR	(26,8894)	(26, 8894)		(0, 676)		(0, 0)	
	*P* value^b^	0.047	0.047	–	0.368	–	1.000	–

^a^Compared to those in the GST group. Redu, reduction = (worm burden median in GST-worm burden median in the experiment group)/worm burden median in GST group × 100%. ^b^The Mann-Whitney *U* test was used to compare the worm burden median. *P* value less than 0.05 means that reduction is significant between GST and experimental groups using median analysis. The meaning of ^a, b^is the same in the following tables

### Protein expression and purification

The expression and purification of EgM4, EgM9 and EgM123 fused with GST (EgM4-GST, EgM9-GST and EgM123-GST) have been described in our previous studies [6, 7]. To test antibody levels, we inserted the EgM genes into the pET28 vector (Novagen). The recombinant vectors were then transformed into *E. coli* (BL21 strain) to express EgM4, EgM9 and EgM123 fused with a 6-His tag. The proteins were purified using affinity columns (Novagen) and then coated onto ELISA plates with 100 μl/well at a concentration of 0.5 μg/ml to measure antibody levels. The details of all methods employed, including serum preparation and the ELISA protocol have been described [7].

### Vaccination and parasite challenge

Two trials were carried out for testing the protective efficacy of the EgM proteins. In trial I, two groups each with seven Beagles were used. One group was vaccinated with EgM9-GST as an experimental group and the other with GST mixed with Quil A as a control group. One dose of vaccine comprised 100 μg of soluble recombinant EgM9-GST or GST and 100 μg of Quil A (Superfos Biosector, Demark) in 250 μl of PBS. The mixture was stirred overnight at 4 °C before vaccination. Trial II used Xinjiang local dogs and involved three groups including Group 1 consisting of 12 dogs vaccinated with GST; Group II consisted of nine dogs vaccinated with EgM4 and Group III had nine dogs vaccinated with EgM123. All proteins were mixed with Quil A. The dogs received one primary vaccination and two booster vaccinations by subcutaneous injections with intervals of 2 weeks between each vaccination.

In addition, we mixed 100 μg of EgM9-GST, EgM123-GST or GST recombinant proteins with 100 μg of ISCOMs (AbISCO− 100, Isconova AB, Sweden) and used the preparation to vaccinate dogs of the Xinjiang local breed.

*E. granulosus* protoscoleces were collected from sheep livers in a slaughterhouse in Urumqi, in western China as previously described [7]. All dogs were orally infected with 200 000 protoscoleces of *E. granulosus* 1 week after the second booster injection.

All the dogs in the above three trials were euthanized and necropsied 45–46 days after infection for collecting and counting *E. granulosus* worms as described in detail previously [7].

### Longevity trial and fecal egg counts

For the longevity trial, two experimental groups of Beagles were vaccinated with EgM9 (*n* = 9) and EgM123 (*n* = 10) mixed with Quil, using the same dose and schedule as above. Two weeks after the last vaccination boost, all dogs were orally challenged with 200 000 protoscoleces. Control dogs (*n* = 10) were vaccinated with only Quil A in PBS. At 40 days post-challenge infection, dog fecal samples were collected every 2 days for the first month and then, every 4 days until 169 days post-infection. To count the number of *E. granulosus* eggs, 3–5 g of fresh dog faeces was weighed and placed into a Falcon tube. Three glass balls (6 mm in diameter) and 25 ml saline were added to the tube. After shaking the tube for 1 min, the faecal sample was passed through a 100 mesh sieve and rinsed with 50 ml saline. The run-through was centrifuged at 1500 g for 10 min. After discarding the supernatant, the pellet was washed three times with 50 ml saline and centrifuged at 1500 g for 10 min. The pellet was then re-suspended in 45 ml of saturated sucrose (1300 g of sucrose in 1000 ml of water) to float the *E. granulosus* eggs. After 90 min at room temperature, the top 30 ml of the supernatant was transferred into a fresh Falcon tube containing 20 ml of water. The tube was then centrifuged at 3000 g for 10 min to sediment the eggs. After washing twice with 45 ml saline and centrifugation at 1500 g for 10 min, the pellet was resuspended in 10 ml of saline. Egg numbers were counted using a McMaster slide by adding 0.15 ml per chamber. Ten chambers were counted for each of the dog faecal samples containing high number of eggs and the whole preparation was used to count eggs in each stool sample with low or no eggs. Eggs per gram (EPG) of dog faeces were used in statistical analysis. All dogs were euthanized and necropsied at day 169 or 170 post-infection.

### Data collection and analysis

The Mann-Whitney U test was used to compare worm burdens and eggs per gram (EPG) in experimental and control groups using SPSS software (release 10.0; SPSS, USA). We used median value in each of the groups (inter-quartile range, IQR) for calculation of reduction in worm numbers and eggs. Spearman’s rank correlation was used to analyse the correlation between worm burden and serum optical-density values in ELISA. *P* < 0.05 was taken to indicate a statistically significant difference.

## Results and discussion

### Vaccine efficacy of EgM proteins combined with Quil A

In trial I, we vaccinated dogs with EgM9 fused with GST mixed with Quil A and we used recombinant GST mixed with Quil A as a control. No significant differences were evident in worm burdens between EgM9 vaccinated dogs and the GST control vaccinated dogs (*P* > 0.05). However, in the control group 16.5% (range from 7.5% to 22.7%) of worms developed to the mature adult stage, compared with only 0.8% (range from 0% to 1.6%) of worms developing to the mature stage in the experimental group. Fewer worms (17.1%) developed 4 segments in the EgM9 vaccinated dogs compared with, 41.1% of the worms with 4 segments in the control dogs (*P* = 0.004; Table 1). The EgM9-GST vaccine induced significant protective efficacy in terms of inhibition of worm growth and suppression of egg production after 45 days post-challenge infection compared with the control dogs.

We tested another two recombinant proteins, EgM4 and EgM123, mixed with Quil A as adjuvant in a second vaccine trial using a local breed of dog from Xinjiang. EgM123-GST induced significant protective efficacy in terms of worm burden reduction and suppression of worm growth and egg production at day 45 post-infection. No worms developed to the 4 segment stage and no worms produced eggs. In contrast, 42.5% of the worms from the control dogs had 4 segments and 16.1% of the worms harboured eggs (Table 2). Similar to our previous study [7], dogs vaccinated with EgM4-GST did not induce any significant protective efficacy (Table 2).

Similar to when emulsified with Freund’s adjuvant, the EgM9-GST and EgM123-GST recombinant proteins mixed with Quil A induced a significant level of protective efficacy in Beagle dogs against *E. granulosus* infection at day 45 post-challenge infection in terms of inhibiting worm growth and suppressing egg production. This supports EgM9 and EgM123 as being encouraging candidates for future vaccine development against *E. granulosus* infection in dogs. As the egg stage is the primary cause of echinococcosis in humans and animals, suppression of egg production in canine definitive hosts will reduce or prevent echinococcosis transmission.

**Fig. 1:**
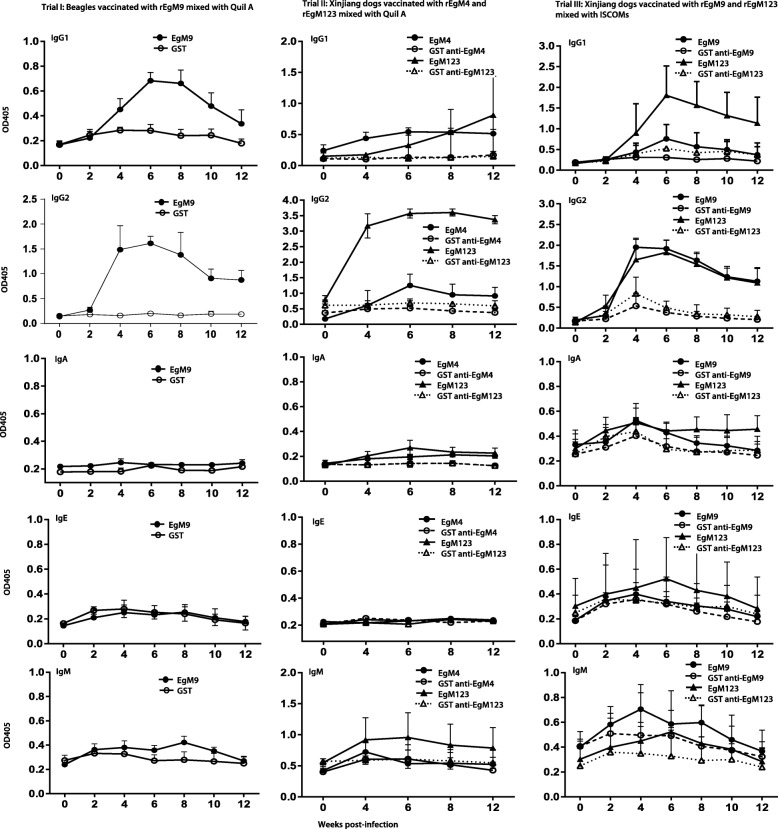
Serum levels of immunoglobulins and subclasses of IgG against recombinant EgM proteins in ELISA. Serum was diluted 1 in 400

Also, similar to Freund’s adjuvant, the EgM proteins mixed with Quil A stimulated a significant IgG1 and IgG2 response (Fig. 1), indicating IgG responses induced by EgM are adjuvant-independent. IgG2 is likely an antibody associated with host protection against infection. However, the antibody levels dropped quickly after challenge infection to a low level at week 6 post-infection, which is the time of reproductive organ maturation and egg production. Determination of the correlation between IgG levels and worm burdens may help in an understanding of the mechanisms underpinning the elicited protective efficacy against worm infection in dogs.

### Vaccine efficacy of EgM proteins combined with ISCOMs

To determine whether ISCOMs could increase the protective efficacy, we mixed them with EgM9-GST and EgM123-GST and immunized dogs of the local breed purchased from Xinjiang villages. No worms developed to the mature stage in either the experimental or control groups by day 45 post-infection. However, EgM123-GST reduced significantly the worm burden (*P* < 0.05) compared with the worm burden in the control dogs (Table 3). The worm burden in the EgM9 group were reduced by 47% (median data analysis) compared with the control group. However, statistical analysis showed no significant difference between the EgM9-GST vaccinated group and the GST vaccinated control group (Table 3). EgM9 andrEgM123 combined with the ISCOMs also induced an IgG2- predominant response (Fig. 1).

ISCOMs are a type of adjuvant that can enhance protective immunity by antigen presentation. We hypothesized that the protection induced by the EgM proteins against *E. granulosus* infection in dogs could be increased by using ISCOMs as an adjuvant. However, in the trial no worms developed to the mature stage in either the experimental or control groups after 45 days post-infection. It is possible that ISCOMs may induce protective efficacy non-specifically resulting in inhibited worm growth.

### Vaccine longevity study

We also performed a vaccine longevity study to test the duration of protection induced by the EgM proteins. The numbers of *E. granulosus* eggs were counted and recorded using 3–5 g of dog faeces. We trialled several methods for counting fecal eggs, including a brine flotation method, but found that the method of flotation of *E. granulosus* eggs with saturated sucrose was repeatable and reliable (data not shown) and was used in the vaccine longevity trial.

We counted egg numbers in 3–5 g of faecal samples collected from day 40 post challenge infection (p.i.). *E. granulosus* eggs did not appear until 49 day p.i in two dogs from the PBS/Quil A group, two dogs from the EgM9/Quil A group, and one dog from the EgM123/Quil A group (Additional file 2: Table S1). From day 51 and day 53 p.i, we did not find any eggs in any of the dog faecal l samples collected. However, in some dogs eggs re-appeared on day 55 p.i; seven out of ten dogs in the PBS/Quil A group released eggs ranging from 0.75 EPG to 436 EPG with an average EPG of 77, two dogs released eggs (266 EPG in one dog and 1 EPG in the other) in the EgM123/Quil A group and one dog in the EgM9/Quil A group had an EPG of 0.3 (Additional file 2: Table S1).

**Fig. 2:**
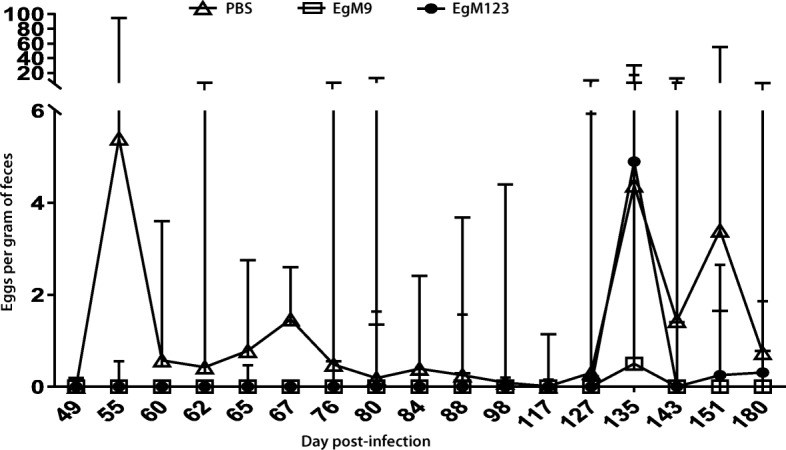
A time course of eggs per gram (EPG) in the faeces of dogs vaccinated with EgM9 and EgM123 compared with PBS against *E. granulosus*

Over the entire time course of egg release from the dogs, there were three peaks (Fig. 2, Additional file 2: Table S1). The first occurred over the period day 55–62 p.i., the second occurred at days 65–76 p.i and the third was between day 127 and day 151 p.i. However, the egg counts were highly variable both daily and between dogs. Considering that environmental pressure is due to eggs being released from dogs, we summed all egg counts from each dog as one sample and compared the EPG counts in the two experimental groups with those in the control group, then analyse the differences by Mann-Whitney *U* test. The analysis showed lower EGP counts in EgM9/Quil A group (*P* < 0.05), whereas EgM123/Quil A group had no difference (*P* > 0.05) compared with the counts in control group. However, we found that EgM123/Quil A group released less eggs before 127 days p.i, (*P* < 0.05), indicating that this protein has partial protection efficacy in term of egg suppression.

## Conclusions

EgM123 and EgM9 mixed with Quil A adjuvant resulted in a similar level of immunogenicity to these proteins emulsified with Freund’s adjuvants, and induced similar levels of protective efficacy in dogs against *E. granulosus* challenge infection. These outcomes reinforce the suitability of EgM123 and EgM9 as vaccine candidates against *E. granulosus* in dogs.

## Additional files

Additional file 1: Multilingual abstract in the five official working languages of the United Nations. (PDF 522 kb)

Additional file 2: **Table S1.** A time course of eggs pre gram (EPG) in the faeces of dogs vaccinated with EgM9 and EgM123 compared with PBS against *E. granulosus*. (XLS 234 kb)

## Abbreviations

IQR: Interquartile range
ISCOMs: Immunostimulating complexes
