# Supplementary material for: Dog vaccination with EgM proteins against Echinococcus granulosus
Source: Infect Dis Poverty. 2018 Jun 13;7:61. doi: 10.1186/s40249-018-0425-4 (PMC5998577; doi:10.1186/s40249-018-0425-4)
Supplement: Supplementary file 1 — Multilingual abstract in the five official working languages of the United Nations. (PDF 522 kb) [file 40249_2018_425_MOESM1_ESM.pdf]

Translation of the abstract into the five official working languages of the United Nations

تطعيم الكلاب ببروتين الـ EgM ضد داء المشوكات الحبيبية

Zhuan-Zhi Zhang, Gang Guo, Jun Li, Bao-Xin Shi, Li Zhao, Bao-Ping Guo, Xu Zhang, Jun-Wei Wang, Xue-Ting Zheng, Wen-Jing Qi, Li He and Wen-Bao Zhang

الملخص

الخلفية: تلعب الكلاب دورا محوريا في نقل داء المشوكات السنخي(CE)، وهو داء حيواني تسببه دودة شريطية (داء المشوكة الحبيبية) ولقد عرضنا سابقا أن الكلاب يتم تطعيمها بطريقتين، حبوب بروتينات خاصة بالديدان البالغة الـ EgM9 والـ EgM123 مستحلب مع (مساعد فريوند) من أجل حماية فعالة بشكل كبير للحد من عبء الدودة وإنتاج البيض بعد 45 يوما بعد الإصابة. ولم يُعرف ما إذا كان يمكن الحفاظ على هذه الحماية باستخدام مواد مساعدة مناسبة للاستخدام في الكلاب.

الطرق: يجب خلط EgM9 وEgM123 مع Quil A أو ISCOMs لتلقيح الكلاب. وبعد ثلاثة عمليات تلقيح، كل الكلاب تأثرت ب 200,000 جرعة من الـE. granulosus. بعد 45 يوما من الإصابة، ماتت كل الكلاب موتا رحيما وتم تشريحها لجمع وتعداد ديدان E. granulosus. المناعية، وتضمن الكشف الـ IgG وفروعها الـ IgG1 وIgG2 للكشف عن مصل الكلاب الملقحة بواسطة ELISA. لتحديد امكانية الحفاظ على فعالية الحماية بعد 45 يومًا بعد الإصابة، طبقنا التجربة العمرية لحساب عدد البيض في براز الكلاب لمدة 170 يومًا بعد الإصابة.

النتائج: الكلاب التي تم تطعيمها مع EgM9 وEgM123 مخلوطا مع Quil A وISCOMs أظهروا حماية ذات فعالية مماثلة لتلك الموجودة في البروتين المستحلب مع مساعد Freund’s في دراستنا السابقة المتعلقة بالحد من الديدان والبيض في 45 يوم بعد العدوى. اظهرت تجربة العمرية ان تطعيم بروتين EgM9 يظهر انخفاض عدد البيض لكل غرام مقارنة بأعداد البيض خلال دراسة سيطرة الكلاب طوال فترة الدراسة.

استنتاج: EgM9 وEgM123 هي عبارة عن بروتينات لقاحات مناسبة ضد داء المشوكات السنخي المعدي في الكلاب.

Translated from English version into Arabic by Mohammad Aljarrah and Abdessalam AIT TOUIJAR, through

TRANSLATORS
WITHOUT BORDERS

EgM 蛋白免疫犬抗细粒棘球绦虫研究

Zhuan-Zhi Zhang, Gang Guo, Jun Li, Bao-Xin Shi, Li Zhao, Bao-Ping Guo, Xu Zhang, Jun-Wei Wang, Xue-Ting Zheng, Wen-Jing Qi, Li He and Wen-Bao Zhang

摘要

引言：囊型包虫病是由细粒棘球绦虫（*Echinococcus granulosus*,E.g）引起的人兽共患病，犬对该病的传播起着至关重要的作用。我们前期试验表明 E.g 成虫特异蛋白 EgM9 和 EgM123 与弗氏佐剂乳化后免疫犬，原头蚴感染 45 日后剖检表明犬产生了明显的抑虫和减卵效果。本研究确定 EgM 蛋白与 Quil A 或 ISCOMs 混合使用能否诱导犬产生抗虫保护效果。

方法：用重组 EgM9 和 EgM123 与 Quil A 或 ISCOMs 混合后免疫犬。经 3 次免疫注射后，每犬口服 20 万个 E.g 原头蚴。感染 45 日后将所有犬安乐处死并剖检计数肠道虫体和

排卵数量，使用 ELISA 方法检测犬血清中的 IgG 抗体及亚类 IgG1 及 IgG2。为确定感染犬 45 天后保护效果是否仍然能够维持，我们延长观察和计数每克犬粪的虫卵时限至感染后 170 天。
结果：EgM9 或 EgM123 混合 Quil A 或 ISCOMs 接种犬，感染 45 天后剖检犬，结果表明接种犬产生了与弗氏佐剂乳化疫苗相似的抑虫和减卵保护效果；保护期实验表明，在整个实验周期内 EgM9 免疫组相对于对照组每克犬粪有更少的排卵量。
结论： EgM9 和 EgMl23 是用于接种犬产生抗细粒棘球绦虫疫苗研制的候选蛋白，Quil A 是可用的佐剂。

Translated from English version into Chinese by Zhuan-Zhi Zhang,

Vaccination canine contre Echinococcus granulosus avec des protéines EgM

Zhuan-Zhi Zhang, Gang Guo, Jun Li, Bao-Xin Shi, Li Zhao, Bao-Ping Guo, Xu Zhang, Jun-Wei Wang, Xue-Ting Zheng, Wen-Jing Qi, Li He et Wen-Bao Zhang

Résumé
Contexte : Les chiens jouent un rôle central dans la transmission de l'échinococcose kystique, une zoonose causée par le cestode *Echinococcus granulosus*. Nous avons précédemment démontré que la vaccination de chiens avec deux protéines spécifiques du ver *E. granulosus* adulte, EgM9 et EgM123, émulsifiées avec des adjuvants de Freund, induisait une protection significative, du point de vue de la réduction de la charge de vers et de la production d'œufs, 45 jours après l'infestation. Cependant, nous ne savions pas si cette protection pouvaient être prolongée à l'aide d'adjuvants pouvant être administrés aux chiens.
Méthodes : Des protéines EgM9 et EgM123 de recombinaison ont été mélangées à du Quil-A® ou à des ISCOM pour la vaccination de chiens. Après trois injections de vaccin, tous les chiens ont été infestés par voie orale avec 200 000 protoscolex d'*E. granulosus*. Quarante-cinq jours après l'infestation, tous les chiens ont été euthanasiés et autopsiés afin de recueillir et de compter les spécimens d'*E. granulosus*. Des immunoglobulines, notamment les sous-classes IgG1 et IgG2, ont été détectées par ELISA dans le sérum des chiens vaccinés.Afin de déterminer si l'efficacité de la protection pouvait être prolongée au-delà de 45 jours après l'infestation, nous avons mis en place un essai de longévité consistant à compter les œufs dans les déjections des chiens pendant 170 jours après l'infestation.
Résultats : Les chiens vaccinés avec de l'EgM9 et de l'EgM123 mélangées à du Quil-A et à des ISCOM présentaient une protection similaire à celle conférée par les protéines émulsifiées avec des adjuvants de Freund dans notre étude précédente, du point de vue de la réduction des vers et des œufs 45 jours après l'infestation. L'essai de longévité a montré que le groupe vacciné avec la protéine EgM9 excrétait moins d'œufs par gramme que les chiens-témoins de l'étude canine.
Conclusion : Les protéines EgM9 et EgM123 constituent par conséquent de bonnes candidates pour un vaccin canin contre *E. granulosus*.

Translated from English version into French by Suzanne Assenat and Rodolphe Blet, through

Вакцинация собак белками EgM для защиты от эхинококка Echinococcus granulosus

Чжуань-Чжи Чжан, Ган Го, Цзюнь Ли, Бао-Синь Ши, Ли Чжао, Бао-Пин Го, Сюй Чжан, Цзюнь-Вэй Ван, Сюэ-Тин Чжэн, Вэнь-Цзин Ци, Ли Хэ и Вэнь-Бао Чжан

Аннотация

Краткая информация. Собакам принадлежит очень важная роль в распространении кистозного эхинококкоза (КЭ). Этот зооноз вызывается ленточным гельминтом из группы цестод *Echinococcus granulosus*. Ранее мы продемонстрировали, что у собак, которых вакцинировали двумя специфическими белками EgM9 и EgM123, полученными от взрослой особи *E. granulosus* и эмульгированными адъювантом Фрейнда, значительно возрастала эффективность защиты, а именно: через 45 дней после инфицирования уменьшалось количество гельминтов и выработка ими яиц. Оставалось неизвестным, можно ли поддерживатьэту защиту, применяя подходящие для собак адъюванты.

Методы. Для вакцинации собак рекомбинантные белки EgM9 и EgM123 смешивали с Quil A или комплексами ISCOM. После трёх инъекций вакцины всех собак подвергли пероральному тестируемому инфицированию в дозе 200 000 протосколексов *E. granulosus*. Через 45 дней после инфицирования всех собак умертвили и провели вскрытие с целью сбора и подсчёта гельминтов *E. granulosus.* При помощи метода ELISA в сыворотке вакцинированных собак были обнаружены иммуноглобулины, в том числе подклассов IgG и IgG2. С целью определения продолжительности сохранения эффективной защиты через 45 дней после инфицирования мы провели долгосрочное исследование с подсчётом яиц гельминта в кале собак на протяжении 170 дней после инфицирования.

Результаты. У собак, вакцинированных смесью EgM9 и EgM123 с Quil A и ISCOM, после введения белков, эмульгированных адъювантом Фрейнда, отмечена эффективность защиты, аналогичная нашему предыдущему исследованию с данными об уменьшении количества гельминтов и яиц через 45 дней после инфицирования. Результаты долгосрочного исследования показали, что вакцинированная белком EgM9 группа собак выделяла меньше яицгельминтов в расчёте на грамм по сравнению с количеством яиц у собак контрольной группы.

Выводы. EgM9 и EgM123 являются подходящими кандидатами для вакцин, направленных на защиту собак от инфекции *E. granulosus.*

Translated from English version into Russian by Ann Nosova and Natalia Potashnik, through

Vacunas para perros con proteínas EgM para luchar contra la Echinococcus granulosus

Zhuan-Zhi Zhang, Gang Guo, Jun Li, Bao-Xin Shi, Li Zhao, Bao-Ping Guo, Xu Zhang, Jun-Wei Wang, Xue-Ting Zheng, Wen-Jing Qi, Li He y Wen-Bao Zhang

Resumen

Antecedentes: Los perros desempeñan un papel fundamental en la transmisión de la equinococosis cística, una zoonosis causada por la tenia *Echinococcus granulosus*. Ya demostramos que los perros vacunados con dos proteínas *emulsionadas con adyuvantes de Freund* de tenia adulta *E. granulosus*, la EgM9 y la EgM123, indujeron una considerable eficacia de protección en cuanto a la reducción de la enfermedad parasitaria y la producción de huevos los 45 días posteriores a la infección. No se conocía si esta protección se podía conseguir al usar adyuvantes apropiados para perros.

Metodología: Los recombinadores EgM9 y EgM123 se mezclaron con las vacunas para perros Quil A o ISCOM. Después de ser vacunados tres veces, se infectó oralmente a todos los perros con 200.000 protoescólices de *E. granulosus*. 45 días después de la infección, todos los perros fueron eutanasiados y se les realizó una necropsia para recolectar y contar gusanos de *E. granulosus*. En el suero sanguíneo de aquellos perros vacunados se detectaron anticuerpos, incluidas las subclases IgG, IgG1 e IgG2, gracias al método ELISA. Para determinar si la eficacia de la protección se mantenía durante los siguientes 45 días a la infección, realizamos una prueba de longevidad para contar los huevos en las heces de los perros durante 170 días después de la infección.

Resultados: Los perros vacunados con EgM9 y EgM123 mezcladas con Quil A e ISCOM mostraron una eficacia de protección similar a la de aquellas proteínas emulsionadas con adyuvantes de Freund de nuestro estudio previo a la hora de reducir gusanos y huevos los 45 días posteriores a la infección. La prueba de longevidad mostró que los grupos de proteínas EgM9 vacunadas expulsaron un número muy bajo de huevos por gramo comparado con aquellos huevos contados en los perros de control durante el estudio de perros.

Conclusiones: La EgM9 y la EgM123 son por lo tanto adecuadas para los aspirantes a la vacuna contra la infección causada por la *E. granulosus* en perros.

Translated from English version into Spanish by Gonzalo de la Orden and Julia.S.Osma, through

TRANSLATORS
WITHOUT BORDERS
